# Supplementary material for: A UMLS-based spell checker for natural language processing in vaccine safety
Source: BMC Med Inform Decis Mak. 2007 Feb 12;7:3. doi: 10.1186/1472-6947-7-3 (PMC1805499; doi:10.1186/1472-6947-7-3)
Supplement: Additional file 13 — RAP application directory. Sets up directory for RAP (RDF application for PHP) source code [file 1472-6947-7-3-S13.gz › rap/doc/phpdoc/__filesource/fsource_default__vocabularyVCARD.php.html]

xml version="1.0" encoding="iso-8859-1"?


File Source for VCARD.php


# Source for file VCARD.php

Documentation is available at VCARD.php

```
1. <?php
2. // ----------------------------------------------------------------------------------
3. // vCard profile defined by RFC 2426 - Vocabulary
4. // ----------------------------------------------------------------------------------
5. // Version                   : 0.9
6. // Authors                   : Daniel Westphal (dawe@gmx.de)
7. //
8. // Description               : Wrapper, defining resources for all terms of the
9. //                               vCard Vocabulary. For details about vCard see: http://www.w3.org/TR/vcard-rdf .
10. //                                Using the wrapper allows you to define all aspects of
11. //                             the vocabulary in one spot, simplifing implementation and
12. //                             maintainence. Working with the vocabulary, you should use
13. //                             these resources as shortcuts in your code.
14. //
15. // ----------------------------------------------------------------------------------
16. // History:
17. // 04-04-2003                 : $VCARD_UID added. Chris Bizer (chris@bizer.de)
18. // 02-21-2003                 : Initial version
19. // ----------------------------------------------------------------------------------


23. // VCARD concepts

25. $VCARD_UID = new Resource(VCARD_NS.'UID');
26. $VCARD_ORGPROPERTIES = new Resource(VCARD_NS . 'ORGPROPERTIES');
27. $VCARD_ADRTYPES = new Resource(VCARD_NS . 'ADRTYPES');
28. $VCARD_NPROPERTIES = new Resource(VCARD_NS . 'NPROPERTIES');
29. $VCARD_EMAILTYPES = new Resource(VCARD_NS . 'EMAILTYPES');
30. $VCARD_TELTYPES = new Resource(VCARD_NS . 'TELTYPES');
31. $VCARD_ADRPROPERTIES = new Resource(VCARD_NS . 'ADRPROPERTIES');
32. $VCARD_TZTYPES = new Resource(VCARD_NS . 'TZTYPES');
33. $VCARD_Street = new Resource(VCARD_NS . 'Street');
34. $VCARD_AGENT = new Resource(VCARD_NS . 'AGENT');
35. $VCARD_SOURCE = new Resource(VCARD_NS . 'SOURCE');
36. $VCARD_BDAY = new Resource(VCARD_NS . 'BDAY');
37. $VCARD_REV = new Resource(VCARD_NS . 'REV');
38. $VCARD_SORT_STRING = new Resource(VCARD_NS . 'SORT_STRING');
39. $VCARD_Orgname = new Resource(VCARD_NS . 'Orgname');
40. $VCARD_CATEGORIES = new Resource(VCARD_NS . 'CATEGORIES');
41. $VCARD_N = new Resource(VCARD_NS . 'N');
42. $VCARD_Pcode = new Resource(VCARD_NS . 'Pcode');
43. $VCARD_Prefix = new Resource(VCARD_NS . 'Prefix');
44. $VCARD_PHOTO = new Resource(VCARD_NS . 'PHOTO');
45. $VCARD_FN = new Resource(VCARD_NS . 'FN');
46. $VCARD_Suffix = new Resource(VCARD_NS . 'Suffix');
47. $VCARD_CLASS = new Resource(VCARD_NS . 'CLASS');
48. $VCARD_ADR = new Resource(VCARD_NS . 'ADR');
49. $VCARD_Region = new Resource(VCARD_NS . 'Region');
50. $VCARD_GEO = new Resource(VCARD_NS . 'GEO');
51. $VCARD_Extadd = new Resource(VCARD_NS . 'Extadd');
52. $VCARD_GROUP = new Resource(VCARD_NS . 'GROUP');
53. $VCARD_EMAIL = new Resource(VCARD_NS . 'EMAIL');
54. $VCARD_Family = new Resource(VCARD_NS . 'Family');
55. $VCARD_TZ = new Resource(VCARD_NS . 'TZ');
56. $VCARD_NAME = new Resource(VCARD_NS . 'NAME');
57. $VCARD_Orgunit = new Resource(VCARD_NS . 'Orgunit');
58. $VCARD_Country = new Resource(VCARD_NS . 'Country');
59. $VCARD_SOUND = new Resource(VCARD_NS . 'SOUND');
60. $VCARD_TITLE = new Resource(VCARD_NS . 'TITLE');
61. $VCARD_MAILER = new Resource(VCARD_NS . 'MAILER');
62. $VCARD_Other = new Resource(VCARD_NS . 'Other');
63. $VCARD_Locality = new Resource(VCARD_NS . 'Locality');
64. $VCARD_Pobox = new Resource(VCARD_NS . 'Pobox');
65. $VCARD_KEY = new Resource(VCARD_NS . 'KEY');
66. $VCARD_PRODID = new Resource(VCARD_NS . 'PRODID');
67. $VCARD_Given = new Resource(VCARD_NS . 'Given');
68. $VCARD_LABEL = new Resource(VCARD_NS . 'LABEL');
69. $VCARD_TEL = new Resource(VCARD_NS . 'TEL');
70. $VCARD_NICKNAME = new Resource(VCARD_NS . 'NICKNAME');
71. $VCARD_ROLE = new Resource(VCARD_NS . 'ROLE');

73. ?>
```

Documentation generated on Fri, 17 Dec 2004 16:18:15 +0100 by phpDocumentor 1.3.0RC3
